# Impacts of the Co-Pyrolytic Product from Waste Cooking Oil (WCO) and Polypropylene (PP) on Physical and Rheological Properties of Bitumen

**DOI:** 10.3390/polym18040475

**Published:** 2026-02-13

**Authors:** Neslihan Atasağun

**Affiliations:** Department of Civil Engineering, Faculty of Engineering and Natural Sciences, Konya Technical University, 42250 Konya, Türkiye; natasagun@ktun.edu.tr

**Keywords:** bitumen modification, asphalt binder modification, co-pyrolysis, storage stability, pyrolytic product

## Abstract

This paper aims to investigate the effects of the co-pyrolytic product produced from the co-pyrolysis of waste cooking oil (WCO) and polypropylene (PP) on pure bitumen by using some physical and rheological tests. To reach this goal, the product was obtained by producing from the co-pyrolysis of WCO and PP at distinct conditions. Different pyrolytic products with different structural properties can be obtained from the co-pyrolysis of various materials at different pyrolysis conditions. It was not found any study in which bitumen was modified with the co-pyrolytic product produced from the co-pyrolysis of WCO and PP materials at specified blending ratios and conditions, as described in this paper. For this reason, this paper investigates the effects of this co-pyrolytic product as an additive on bitumen in order to improve some of the rheological and physical properties of bitumen and to overcome some problems for the first time. The mixture ratio was determined as 1:2 (WCO:PP). PG 64-22 neat bitumen was modified with this co-pyrolytic product, and some features of the bituminous binders were detected by using differential scanning calorimetry (DSC), Fourier transform infrared spectroscopy (FTIR), penetration, softening point, dynamic shear rheometer (DSR), rotational viscometer (RV), a rolling thin film oven test (RTFOT), a pressurized aging vessel (PAV), a bending beam rheometer (BBR), storage stability, and scanning electron microscopy (SEM) tests. From the FTIR results of the modified binders, it was found that the intensity of the peak around 2357.69 cm^−1^ increased with the addition of this pyrolytic product. This pyrolytic additive hardened the pure bitumen’s consistency, increased its viscosity, improved its resistance against rutting deformations, and enhanced its high-temperature performance. It can be said that PG 64-22 pure bitumen can easily be modified with this pyrolytic product at the conditions described in this study. Additionally, this co-pyrolytic product improved the high-temperature performance grade (PG) of pure bitumen from PG 64 to PG 76 when it was used at 5% of the weight of neat bitumen. The findings demonstrated that the modified bituminous binders containing 3% and 5% co-pyrolytic product had suitable storage stabilities.

## 1. Introduction

The modification of bituminous binders may be considered one of the most efficient ways for developing flexible pavement performance [1,2,3,4,5,6,7]. Owing to the increasing traffic loads and changing climatic conditions, pure bitumen cannot provide enough resistance against deformations occurring in flexible pavements. For this reason, many researchers have studied enhancing the properties of bitumen by modifying it with various polymer additives [1,2,3,4,5,6,7,8]. Among them, polypropylene (PP) can be considered one of the most commonly preferred thermoplastic polymer materials used as an additive in bitumen [2,4,6,8,9,10,11]. PP may have some significant mechanical and physical properties for various applications [12,13]. In the literature, different PP materials such as waste PP, recycled PP, raw PP, etc., have been used as additives in bitumen for different ratios and improved the high-temperature properties of bitumen [2,3,4,5,6,7,8,9,10,11].

However, studies in the literature have reported that the storage stability, phase separations [1,2,3,4,5,7,11] and low-temperature properties [2,7,14,15] of the modified bituminous binders with several PP materials for different dosages and various conditions may have critical importance on the performance. The polypropylene melting point is at about 160–175 °C based on its structural properties [2,3,8,12,13]. Some researchers have modified 50/70 or PG 64 pure bitumen with PP at temperatures of approximately 180–190 °C and 2000–5000 rpm [2,3,4,5,7,8] for 120–180 min [3,4,7]. One of the studies in the literature [14] used maleic anhydride to enhance the compatibility of bitumen and PP. The mentioned study [14] carried out the mixing of bitumen and the additive at 170 °C at 5000 rpm, and following this step, an additional mixing was performed [14].

On the other hand, in comparison with studies on modification with various PP materials [2,3,4,5,7,8], the pyrolytic char powders of PP obtained after the grinding process have also been used as additives in bitumen, and these chars have also enhanced the high-temperature performance of bitumen [16,17,18,19,20]. Moreover, modifications of bituminous binders with these PP pyrolytic chars have been carried out at approximately 160 °C and relatively shorter modification durations [16,17,18,19,20] than other raw PP, waste PP, and recycled PP materials [2,3,4,5,7,8].

Easy modification is an important factor for preparing modified bituminous binders. Modification conditions may vary depending on some important factors such as the structural properties of the additives and different bituminous binders [1,2,3,4,5,6,7,8,9,10,14,15,16,17,18,19,20]. Preparing effective modified binders with better storage stability at more applicable modification conditions including mixing speeds, temperatures and modification durations may have importance in terms of both the performance of bituminous binders and economic and environmental effects [1,2,7]. Obtaining the high-temperature performance of raw PP-modified bitumen with easy modification and finding solutions to some problems by using novel additives is important.

In this paper, the co-pyrolysis method was used for the purpose of producing the pyrolytic product used as an additive for bitumen modification. By using the pyrolysis method, valuable liquid, solid and gas products can be obtained. Furthermore, with the co-pyrolysis method, various materials can be pyrolyzed together at the same time, and different pyrolytic products can be obtained at different pyrolysis conditions [21,22,23,24,25,26]. The assorted chemical reactions and synergistic effects may occur as a result of the interactions of different materials during co-pyrolysis, and more quality products can be obtained [21,22,23,24,25,26]. One of the most important parameters which can affect the structure and properties of the pyrolytic products may be pyrolysis temperature [21,22,23,24,25,26,27,28,29,30,31,32]. At relatively higher temperatures, solid carbonaceous chars with different properties can be obtained from different plastics as a result of the various pyrolysis conditions [21,22,30,31,32]. On the other hand, at lower temperatures, different solid pyrolytic products which have different properties can be obtained [23,27,28,29] from other carbonaceous char products [21,22,30,31,32] at higher temperatures from the pyrolysis of several plastic materials. This may be because the materials cannot be further decomposed at lower pyrolysis temperatures [27,28,29]. Additionally, the mixture ratio of different materials can be evaluated as another important parameter of the co-pyrolysis process for obtaining more valuable products. Studies in the literature have shown that different blending ratios of the two materials may affect the properties of the products obtained as a result of the interactions and synergistic effect which occur during the co-pyrolysis process [21,22,23,24,25].

In this paper, it was purposed to investigate the effects of the co-pyrolytic product produced from the co-pyrolysis of WCO and PP at distinct conditions on some properties of pure bitumen. To achieve this aim, firstly, the production of the pyrolytic product was carried out from the co-pyrolysis of WCO and PP in order to use it as an additive in bitumen. Neat bitumen was modified with this pyrolytic product for the first time. Some test methods including DSC, FTIR, RV, penetration, softening point, DSR, RTFOT, PAV, BBR, storage stability and SEM were applied on bituminous binders. In this way, it may be possible to improve some of the rheological and physical properties of bitumen and to overcome some of the problems. It was not found any study in which bitumen was modified with this co-pyrolytic product produced from the co-pyrolysis of WCO and PP materials at the conditions as described in this study. For this reason, this study is an original research work and may have important contributions to the literature.

## 2. Materials and Methods

### 2.1. Materials

In this study, 50/70 (PG 64-22) neat bitumen was used and procured from the Refinery of Kırıkkale City via Konya City Municipality in Türkiye. Some features of neat bitumen and the additive can be seen in Table 1 and Table 2, respectively. The product used as an additive in this research was produced by using the co-pyrolysis method as explained in detail in Section 3.1 of this paper. This co-pyrolytic product was manufactured from the co-pyrolysis of the mixture of WCO and PP materials in certain ratios at distinct conditions. PP buplene 6531 from Lukoil oil company was used as PP material, and WCO was supplied from the University student restaurant.

### 2.2. Methods

In this study, pure bitumen was modified with the co-pyrolytic product in order to determine the effects and potential improvements on some of the rheological and physical properties of bitumen. Firstly, the product was obtained from the co-pyrolysis of WCO and PP at distinct conditions. Neat bitumen was modified with this co-pyrolytic product. Some test methods including DSC, FTIR, RV, penetration, softening point, DSR, RTFOT, PAV, BBR, storage stability and SEM were applied on bituminous binders.

#### 2.2.1. Co-Pyrolysis Experimental Method

**Scheme 1:**
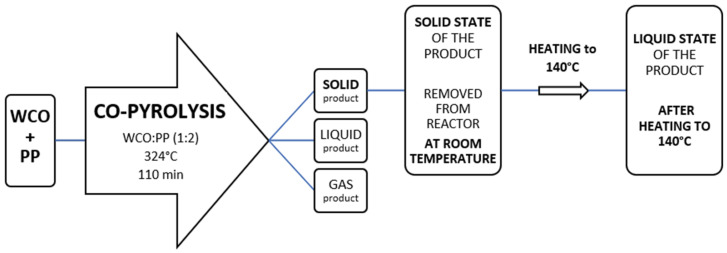
Production of the co-pyrolytic product.

The co-pyrolysis process can be used in order to obtain more valuable products. By using the co-pyrolysis method, different materials can be pyrolyzed together at the same time. During co-pyrolysis, the synergistic effects may occur as a result of various reactions based from the interactions of different materials, and higher-quality products can be obtained [21,22,23,24,25,26]. In this paper, the additive was obtained from the co-pyrolysis of WCO and PP for a certain ratio of 1:2 (WCO:PP) at 324 °C temperature in an oxygen-free environment. Production of the co-pyrolytic product is displayed in Scheme 1.

The pyrolysis temperature can be considered one of the significant factors which can affect the structures and properties of the pyrolytic products [21,22,23,24,25,26,27,28,29,30,31,32]. At lower pyrolysis temperatures, different solid pyrolytic products with different properties [23,27,28,29] can be obtained compared with other carbonaceous char products [21,22,30,31,32] produced at higher temperatures from the pyrolysis of several plastic materials. This may be because the materials cannot be further decomposed at lower pyrolysis temperatures [27,28,29].

Additionally, the mixture ratio which can be considered as another important parameter of the co-pyrolysis process for obtaining more valuable products was determined as 1:2 (WCO:PP) in this paper.

Studies in the literature have shown that different blending ratios of WCO and polyolefin plastics may affect the product properties as a result of the interactions and synergistic effect between two materials during the co-pyrolysis process [23,24,25]. Some studies found in the literature, generally, have investigated the co-pyrolysis of WCO and polyolefin plastic wastes for the purpose of enhancing the properties and yields of liquid pyrolysis products for producing liquid fuels [23,24,25]. One of the studies [23] investigated the co-pyrolysis of WCO and polyolefin plastic wastes (WP) for different blending rates. It has been determined that the highest liquid yield can be obtained for a blending ratio of WCO:WP (1:2) as a result of the synergistic effect [23]. PP may have some important mechanical and physical properties such as hardness, elastic modulus and density for various applications [12,13]. The melting point of the PP, which is one of the polyolefin polymers, is approximately 160–175 °C based on its structural features [2,3,8,12,13]. On the other hand, WCO can be obtained as a result of frying the edible oils repeatedly. WCO may have different compositions and properties based on its origin [23,25,40,41,42]. During WCO decomposition, firstly, the components which have low boiling points below 200 °C may evaporate [41,42]. It has been reported that the triglycerides found in the structure of the WCO can be decomposed to fatty aldehydes and fatty acids below or at about 300 °C [23,25,41,42]. The synergistic effect may occur between the evaporates from the WCO components and polymer materials during the co-pyrolysis process. As a result of these reactions, long hydrocarbon chains may have been broken into shorter hydrocarbon chains [23,25]. As the temperature increases, primary products may occur as a result of the decomposition of polymer. During polymer degradation, hydrogen can be removed from the polymer chain, and synergistic effects may occur with WCO [23,24,25].

#### 2.2.2. Bitumen Modification

Bitumen used in flexible pavements as a binder has been modified with various additives to improve some of its properties. In this way, it can be possible to decrease and overcome some of the deformations occurring in the flexible pavements. Modification conditions may vary depending on some important factors such as the structural properties of the additives and different bituminous binders [1,2,3,4,5,6,7,8,9,10,14,15,16,17,18,19,20].

Easy modification and modification conditions are important factors for preparing modified bituminous binders. Studies in the literature were perused and compared in terms of both the modification conditions of raw PP-modified bituminous binders and the modification conditions of PP char-modified binders.

In this paper, the co-pyrolytic product was produced and used as an additive in bitumen. The purpose of the modification of pure bitumen with this pyrolytic product is to determine the effects of this additive and to detect the improvements regarding some of the rheological and physical properties of bitumen with providing easy modification.

This pyrolytic product used in this paper was removed from the reactor at room temperature in a solid state and heated to 140 °C to obtain the liquid state of this product. The liquid state of the product at 140 °C was used as an additive for the modification of bitumen for the first time.

In this study, PG 64-22 neat bitumen was modified with this pyrolytic product at different ratios including 3 wt.%, 5 wt.%, and 7 wt.% of pure bitumen, as explained in detail in Section 3.2. In this paper, the modification conditions were determined to be 160 °C temperature, 1200 rpm for 45 min after considering the literature. These modification conditions can be evaluated as relatively more practicable compared with some of the studies in the literature [2,3,4,5,7,8], which have carried out the modification of bitumen with various PP materials at 180–190 °C temperatures, 2000–5000 rpm [2,3,4,5,7,8,14] for 120–180 min [3,4,7].

#### 2.2.3. Binder Aging Methods

The influences of heat and air during the construction and mixing of asphalt binders can be simulated by using RTFO tests according to AASHTO T240 [36]. Bituminous binders are exposed to the airflow at 4000 mL/min, and the test temperature is 163 °C as described in the standards. The mass losses of binders as a result of the RTFO test can also be determined [36]. The aging of bituminous binders throughout the long-term service life of the flexible pavement can be simulated by using a PAV test in accordance with the standard [43]. A PAV test can be applied on the residue of RTFOT aged binders. In accordance with the PAV test, bituminous binder samples are exposed to specified temperatures and conditions for 20 h as described in the specifications [43].

#### 2.2.4. Binder Test Methods

FTIR analysis can be evaluated as one of the commonly used techniques for detecting the functional groups in the materials’ structure. The functional groups can be determined from the spectra obtained from vibrations of the molecules as a result of absorbing infrared rays [23,26,44,45]. Some of the thermal features of the bituminous binders and various materials can be evaluated by using a DSC test [8,9,46,47,48]. The consistency of bituminous binders can be assessed in accordance with the penetration [33] and softening point [34] test results, and the viscosity values can be detected by using an RV test according to the ASTM-D4402 [35] specification. The viscoelastic behaviors and performance grades (PGs) of asphalt binders can be assessed by using the DSR test results according to AASHTO T315 [37]. The rutting resistance and fatigue performance of the binders can be evaluated by using G*/sinδ and with G*.sinδ parameters, respectively [37]. The specification criteria of the G*/sinδ rutting parameter is limited to a minimum of 1.0 kPa for unaged binders and 2.2 kPa for RTFOT aged binders [36,37,49]. The thermal cracking performance of bituminous binders at low temperatures can be determined by using a BBR test [38]. According to this test method, the bituminous binder beam described in the standard [38] is subjected to the loading at its midpoint at low temperatures. The creep stiffness and m value can be determined as a result of the BBR test. The creep stiffness and m value can be used to evaluate the low-temperature performance of bituminous binders. The creep stiffness is limited to a maximum of 300 MPa, and the m value is limited to a minimum of 0.3 according to the specifications [39,49].

#### 2.2.5. Storage Stability and SEM Analysis

The compatibility of bitumen with the additives and storage stabilities of the modified bituminous binders can be evaluated according to the EN13399 [50] and EN 14023 [51] standards. The EN13399 test method includes keeping the modified bituminous binders at 180 °C for 3 days. After cooling the binders to the specified temperature according to the standard, they are cut into three equal parts. The storage stabilities of asphalt binders can be evaluated as a result of the penetration and softening point tests performed on the top and bottom parts of the asphalt binders. The differences in softening points between the bottom and top parts of the asphalt binders are limited to 5 °C, and the penetration differences are limited to 9 mm^−1^ [50,51]. Some of the microstructural properties and distributions of additives in asphalt binders can be viewed by using an SEM test [11,52,53]. The storage stability test results were supported with the SEM images in this paper.

## 3. Results and Discussion

In this paper, the aim of the modification of pure bitumen with this pyrolytic product is to investigate the effects of this additive and to determine the potential improvements on some of the rheological and physical properties of bitumen.

Specifically, neat bitumen was modified with this co-pyrolytic product for the purpose of enhancing high and low-temperature performance, improving the PG of pure bitumen, increasing its softening point, decreasing its penetration, and obtaining suitable storage stability and workability with easy modification.

For this reason, firstly, the production of the pyrolytic product was carried out from the co-pyrolysis of WCO and PP in order to use it as an additive in bitumen [Scheme 1]. Some test methods including DSC, FTIR, RV, penetration, softening point, DSR, RTFOT, PAV, BBR, storage stability and SEM were applied on bituminous binders. The test results, discussions and comparisons are presented as the following.

### 3.1. Production of the Co-Pyrolytic Product

**Figure 1:**
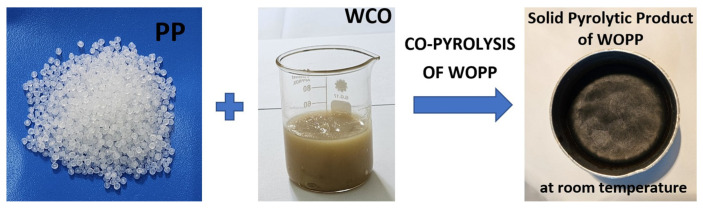
PP, WCO and pyrolytic solid product of WOPP removed from reactor at room temperature.

The pyrolytic product was obtained from the co-pyrolysis of WCO and PP at 324 °C temperature by using nitrogen gas. The mixture ratio was determined as 1:2 (WCO:PP). In this paper, this mixture was referred to as ‘WOPP’. The co-pyrolysis tests of WOPP were performed starting at room temperature and heating up to the desired test temperature (324 °C) in an oxygen-free environment. The oxygen-free environment was supplied by flowing nitrogen gas into the pyrolysis reactor for 15 min when the test started. The pyrolysis reactor was heated up to the test temperature (324 °C), and the co-pyrolysis tests were completed approximately for 110 min. The products were removed after cooling the reactor to room temperature. WCO, PP and the pyrolytic solid product of WOPP removed from the reactor at room temperature are shown in Figure 1.

**Figure 2:**
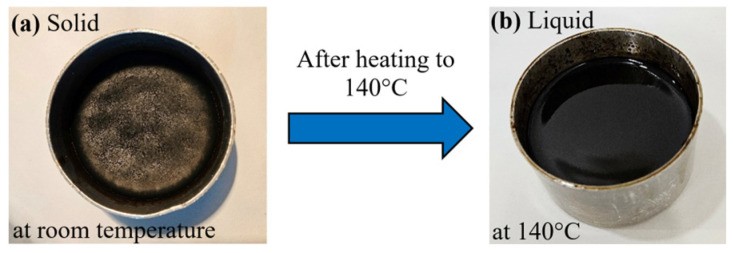
Pyrolytic product of WOPP removed from reactor (WOPPr). (**a**) Solid state of the WOPPr. (**b**) Liquid state of the WOPPr at 140 °C.

In this study, the co-pyrolysis of WOPP was performed at 324 °C temperature, and the blending ratio was determined as 1:2 (WCO:PP) for the aim of obtaining more valuable and a potentially suitable product for bitumen modification. The co-pyrolytic product of WOPP removed from the reactor was referred to as ‘WOPPr’, in this study. This product [Figure 2] may have important structural properties for the modification of asphalt binders. FTIR and DSC tests were applied on the WOPPr pyrolytic product. 

### 3.2. Bitumen Modification with WOPPr

The pyrolytic product removed from the reactor (WOPPr) at room temperature was found in a solid state (Figure 2a), and it was heated to 140 °C to obtain the liquid state of this product. The liquid state of the WOPPr at 140 °C (Figure 2b) was used as an additive in PG 64-22 neat bitumen at different ratios including 3 wt.%, 5 wt.%, and 7 wt.% of pure bitumen. This additive was mixed with the pure bitumen at 160 °C temperature, 1200 rpm for 45 min, considering the literature. It can be said that the pure bitumen can easily be modified with this pyrolytic product at the conditions described in this study. The additive and bituminous binders were defined as follows:

Pure Bitumen: Control binder (unmodified);

WOPPr: Pyrolytic product of WOPP removed from reactor;

3WOPPr: Pure Bitumen + 3 wt.% WOPPr;

5WOPPr: Pure Bitumen + 5 wt.% WOPPr;

7WOPPr: Pure Bitumen + 7 wt.% WOPPr.

### 3.3. FTIR Spectroscopy Test Results

**Figure 3:**
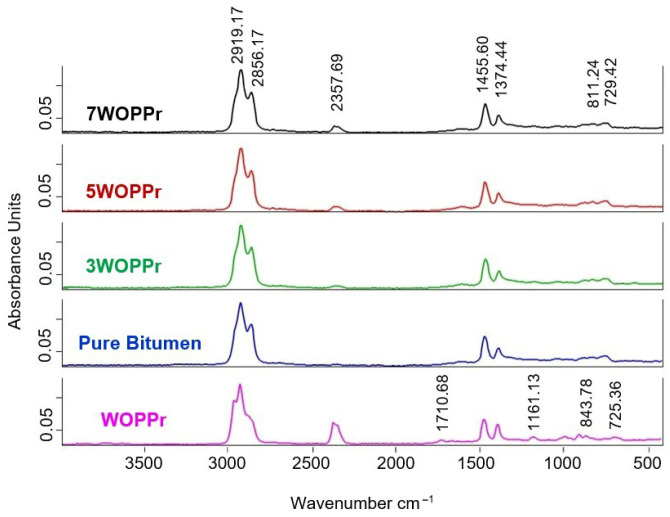
FTIR test results of WOPPr and bituminous binders.

FTIR spectroscopy results were determined as displayed in Figure 3. An increase in the intensity of the peak was observed around 2357.69 cm^−1^ which can be due to the C=O stretching vibrations. In addition, it can also be seen from Figure 3 that the intensity of the peak around 2357.69 cm^−1^ increased as the WOPPr additive content increased. The peaks around 2856.17 cm^−1^ and 2919.17 cm^−1^ can be referred to the C-H stretching vibrations [24,25,26,44,45,53]. The peak seen around 2357.69 cm^−1^ may come from the C=O stretching vibrations [26,42]. The presence of the peak around 1710.68 cm^−1^ can be corresponded to C=O stretching from carbonyl compounds [24,25,26,42,45]. The observed peaks about 1455.60 cm^−1^ and 1374.44 cm^−1^ can indicate the C-H bending vibrations [24,25,26,45]. The peak observed at about 1161.13 cm^−1^ can be based on the stretching of C-O vibrations [14,25,26,42]. The peaks around 843.78 cm^−1^ and 725.36 cm^−1^ may refer to the C-H aromatic bending vibrations [24,26,30,44,45].

### 3.4. Differential Scanning Calorimetry (DSC) Test Results

**Figure 4:**
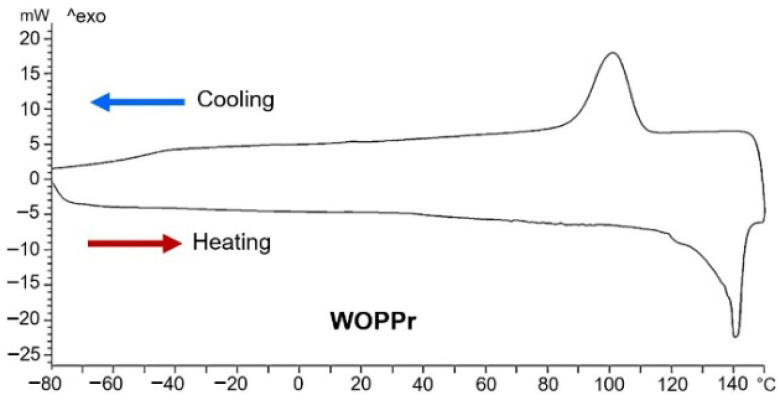
DSC results of the WOPPr.

**Figure 5:**
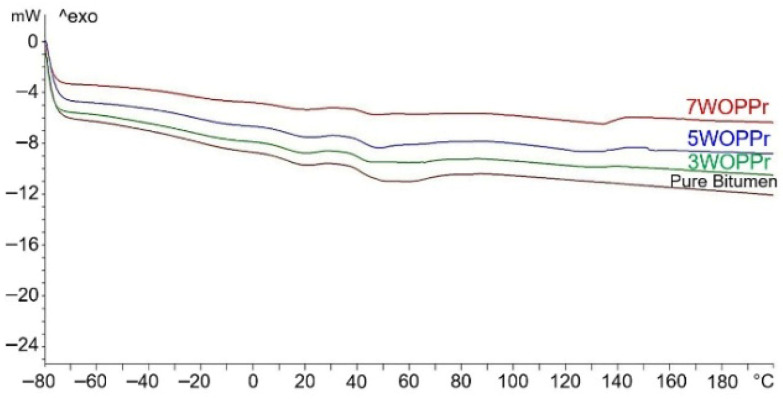
DSC results of the bituminous binders.

The WOPPr pyrolytic product subjected to the DSC test was firstly heated from −80 °C to 150 °C with a 10 °C/min heating rate. Then, the product was cooled from 150 °C to −80 °C with the same rate, and the curves in Figure 4 were obtained. Figure 5 was obtained as a result of the DSC tests applied to bituminous binders. After conditioning at −80 °C for 15 min, the binders were heated from −80 °C to 200 °C temperature with a 10 °C/min heating rate. From Figure 4, the heating curve of the DSC test shows that the melting endothermic peak of the pyrolytic product is at about 140 °C peak temperature. Additionally, from the cooling curve of the DSC test [Figure 4], the exothermic peak of the WOPPr product is at about 101 °C peak temperature. The crystalline degrees and thermal behaviors of various materials can be evaluated by using the DSC test [9,11,46,47,48,54,55]. The crystalline degree of the WOPPr co-pyrolytic product may affect some of the properties of bituminous binders. The effects of the pyrolytic product and thermal behaviors of bituminous binders can be seen in Figure 5. When it comes to the raw PP materials, on the other hand, the melting point of raw PP is approximately 160–175 °C [2,3,7,8,12,13]. One study in the literature [9] showed that different melting peak temperatures were noticed for different PP materials such as 156.1 °C, 154.4 °C and 166.1 °C. Another study [55] reported the melting peak temperatures of different PP materials at 160 °C and 161 °C as a result of the DSC test. The crystalline degree of different kinds of PP materials can affect the bitumen stiffness [9,54,55].

### 3.5. Softening Point and Penetration Test Results

**Figure 6:**
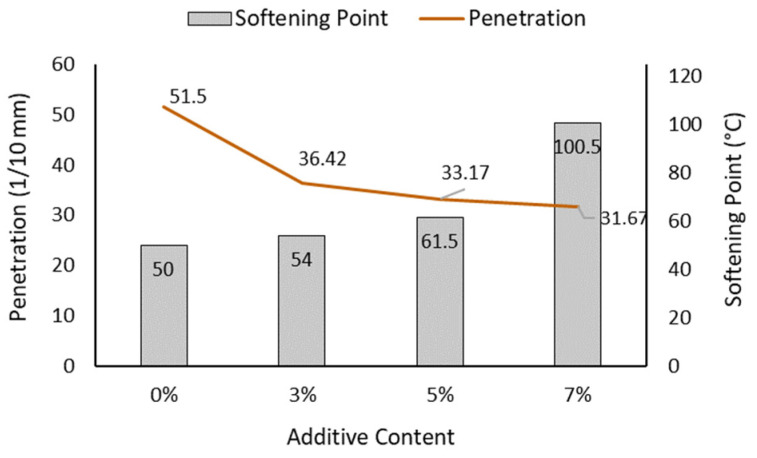
Softening point and penetration values of bituminous binders.

The consistency of the asphalt binder can be determined as a result of the penetration [33] and softening point [34] tests. From the test results seen in Figure 6, it was determined that the penetration value of neat bitumen reduced, while the softening point raised as the amount of pyrolytic product in pure bitumen increased. It can be said that from Figure 6, the WOPPr pyrolytic additive used in this research significantly increased the softening point of pure bitumen when it was used at a rate of 7% by the weight of bitumen. This result may have been obtained as a result of the crystalline degree and structural properties of the co-pyrolytic product used in this paper. Some of the properties of the WOPPr pyrolytic product and modified bituminous binders are displayed in Figure 4 and Figure 5. It was seen from Figure 6 that the softening points of the 3WOPPr and 5WOPPr-modified bituminous binders were approximately 8% and 23% higher than the value of pure bitumen, respectively. In addition, it was determined that the softening point of the 7WOPPr modified bitumen was approximately 2.01 times that of the softening point of neat bitumen. In accordance with Figure 6, it was detected that the penetration values of asphalt binders with 3 wt.%, 5 wt.% and 7 wt.% additives were approximately 29.28%, 35.6% and 38.5% less than that of pure bitumen, respectively.

Comparing with the literature, it can be seen that waste PP [2,10], recycled PP [7], raw PP [4] and PP pyrolytic powder additives [16,17,20] have also hardened the consistency of pure bitumen by reducing its penetration and raising the softening point [2,7]. In one study [4], 5% raw PP additive increased the softening point of PG 64 neat bitumen approximately 8.9% and decreased the penetration value. Another study [17] determined that 5% PP pyrolytic powder additive increased the softening point of 50/60 pure bitumen nearly by 20.9% and decreased the penetration by 41.1%. One study [16] demonstrated that 4%PP pyrolytic powder addition raised the softening point of 40/50 pure bitumen by about 20.4% and reduced the penetration value approximately 30.9%.

### 3.6. Storage Stability Test Results

The storage stability of the modified bituminous binder can be considered one of the most important properties that can influence the performance of flexible pavements. Phase separation problems may occur during storage on account of the density difference between additives and bitumen as a result of the lower compatibilization. Studies in the literature have reported that storage stability and phase separation may be appear in bituminous binders modified with different kinds of PP materials [1,2,3,5,7,19,48].

**Figure 7:**
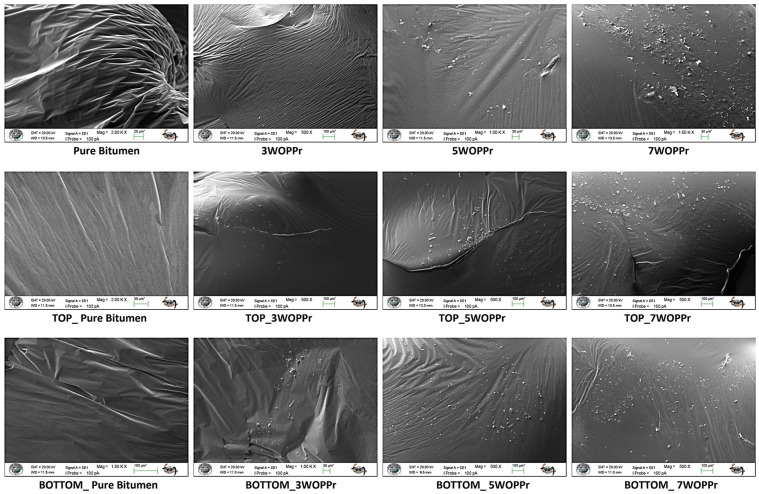
SEM images of bituminous binders.

In this paper, the storage stabilities of the WOPPr-modified bituminous binders were determined according to EN 13399 [50] and EN 14023 [51]. As a result of the penetration and softening point tests carried out on the top and bottom parts of the binders, the storage stability of the binders can be determined. The differences in the softening point values between the top and bottom parts of the asphalt binders in the tubes are limited to a maximum of 5 °C, and the penetration differences are limited to a maximum of 9 mm^−1^ [50,51]. After the storage stability test, from Table 3, it can be seen that the top parts of the modified binders in the tubes have higher softening points and lower penetration values than the bottom parts. This result may have been obtained due to the accumulation of the WOPPr additive at the top parts of the tubes. It was displayed in Table 3 that the penetration differences between the bottom and top parts of the bituminous binders did not exceed the 9 mm^−1^ specification limit. However, when it comes to the softening point differences, it was determined that the 7WOPPr bituminous binder exceeded the specification limit of 5 °C. In accordance with the results, it can be concluded that 3WOPPr and 5WOPPr binders have suitable storage stabilities, while 7WOPPr bituminous binder is not suitable for storage due to exceeding the specification limit of the softening point difference. Additionally, the storage stability test results were supported with the SEM images. The SEM images of bituminous binders before and after applying the storage stability test can be seen in Figure 7.

### 3.7. SEM Analysis Results

SEM images are generally used for observing the microstructure and dispersion of the additives in bitumen [11,52,53]. The dispersion of WOPPr additive in pure bitumen can be viewed by using the SEM images from Figure 7. The softening point and penetration test results before [Figure 6] and after [Table 3] the storage stability tests were supported with SEM images [Figure 7]. Considering the results from the softening point, penetration, DSC and storage stability tests, it was decided to investigate some of the rheological features of pure bitumen and 5WOPPr-modified bitumen by using RV, DSR, RTFOT, PAV and BBR tests.

### 3.8. RV Test Results

Viscosity, a measure of the flow resistance, can be evaluated as one of the important properties of the bituminous binders [1,2,7,49]. The viscosity values of asphalt binders can be determined by using an RV test in accordance with ASTM-D4402 [35] and limited to the specification criteria of 3000 cp at 135 °C [35,49]. From Table 4, it was determined that 5 wt.% WOPPr pyrolytic product additive increased the viscosity of pure bitumen approximately 31.9% and did not exceed the specification limit of 3000 cp at 135 °C. Consequently, according to the specifications [35,49], it was determined that the 5WOPPr binder was suitable in terms of workability. Studies in the literature have shown that waste PP [10], recycled PP [5,8], raw PP [4,53] and PP pyrolytic powder additives [16,17,18] have also increased the viscosity of pure bitumen [2,7].

### 3.9. Binder Aging Test Results

Short-term aged binders according to the RTFO test [36] can be subjected to the PAV test [43,49]. The PAV aging method represents the aging of bituminous binders that occurs during long-term pavement service life [43,49]. The binder specimens after PAV aging can be used for DSR [37] and BBR [38] tests to detect the fatigue performance and low-temperature performance of bituminous binders [37,38,49]. As a result of the RTFO test, mass change must not exceed 1% [39,49]. The results of the RTFO test from Table 5 show that bituminous binders did not exceed the 1% specification limit of mass change. In addition, it can be said that the 5WOPPr-modified bitumen is suitable in terms of the amount of volatile loss that occurred during mixing and construction.

### 3.10. DSR Test Results

A DSR test can be performed on binders in order to assess the rheological properties of the bituminous binders at intermediate and high temperatures. The viscoelastic behaviors and performance grades (PGs) of the binders can be assessed by using the DSR test results [37,49]. The performance of the binder against fatigue and rutting deformations can be determined by using G*.sinδ and G*/sinδ parameters, respectively [37,49]. The specification criteria of the G*/sinδ rutting parameter is limited to a minimum of 1.0 kPa for unaged binders and 2.2 kPa for RTFOT aged binders [36,49]. The higher value of the G*/sinδ, the better resistance to rutting deformations [4,7,49].

**Figure 8:**
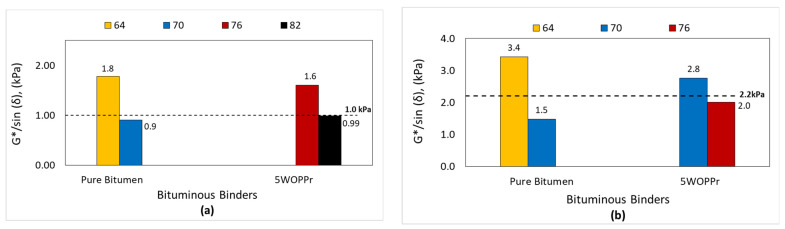
Rutting parameters of bituminous binders: (**a**) unaged binders, (**b**) after RTFOT.

The DSR test results of the unaged and RTFOT aged binders are displayed in Figure 8a and Figure 8b, respectively. According to the DSR tests applied to the unaged binders, it was determined that the pure bitumen and 5WOPPr binders met the minimum 1.0 kPa specification criterion of G*/sinδ at 69.1 °C and at 81.9 °C, respectively [Figure 8a]. When it comes to the DSR test results of RTFOT aged binders, it was determined that neat bitumen and 5WOPPr binders met the minimum 2.2 kPa specification criterion at 67.2 °C and at 74.3 °C, respectively [Figure 8b]. The test results showed that the WOPPr pyrolytic product increased the resistance against rutting deformations and improved the high-temperature performance of pure bitumen.

Comparing with the literature, it was determined that different sources of PP additives [4,8,11] and PP pyrolytic powders [18,19,20] also increased the rutting resistance of bituminous binders [2,7]. One study [4] demonstrated that the modified bitumen containing 5% raw PP met the 1.0 kPa criterion of G*/sinδ at 71.7 °C. Another study [11] determined that 5% raw PP increased the G*/sinδ temperature at 1.0 kPa from 61.5 °C to 69.2 °C.

**Figure 9:**
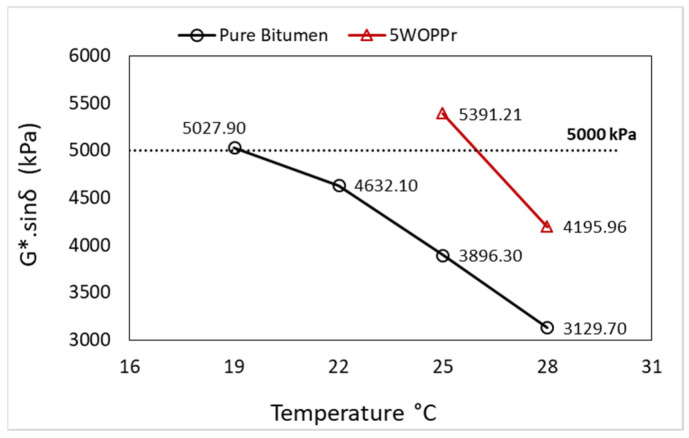
Fatigue parameters of bituminous binders.

From the DSR test after RTFOT and PAV aging, the fatigue parameter values of G*.sinδ were found as shown in Figure 9. The stiffness of the bituminous binder increases as the G*.sinδ parameter rises, and the G*.sinδ fatigue parameter is limited to the maximum 5000 kPa specification criterion [39,49]. It was determined that 5 wt.% WOPPr pyrolytic additive stiffened the pure bitumen and increased the G*.sinδ fatigue parameter approximately 38.4% and 34.1% at 25 °C and 28 °C temperatures, respectively. From Figure 9, pure bitumen and 5WOPPr binders met the maximum 5000 kPa criterion at 22 °C and at 28 °C, respectively. Comparing with the literature, one study [6] showed that the fatigue parameters of the 4% and 6% recycled PP modified bituminous binders exceeded the max. 5000 kPa limit value at 25 °C.

### 3.11. BBR Test Results

A BBR test can be used to assess the low-temperature performance of bituminous binders, in pursuance of AASHTO T313 [38] by using the creep stiffness (S) and the creep ratio (m-value) obtained from the BBR test [38,49]. Due to the fact that bituminous binders are viscoelastic materials, their stiffness increases at low temperatures. The creep stiffness modulus (S) of the binders increases, and the m-value decreases with decreasing temperatures. The creep stiffness is limited to a maximum of 300 MPa, and the m-value is a minimum of 0.3 according to the specifications [39,49].

**Figure 10:**
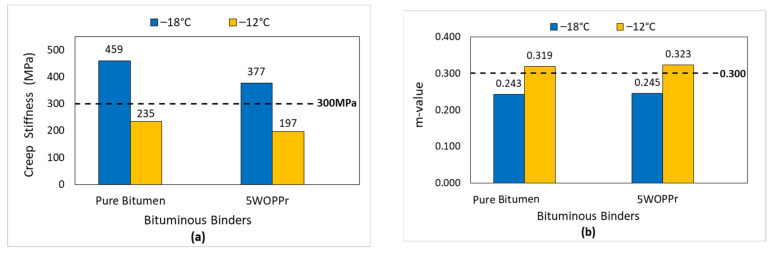
BBR test results of bituminous binders: (**a**) creep stiffness, (**b**) m values.

As can be displayed from Figure 10, the BBR test results have shown that 5 wt.% WOPPr pyrolytic additive reduced the creep stiffness, slightly increased the creep ratio, and positively affected the low-temperature performance of pure bitumen. Comparing with the studies in the literature [14,15], it was determined that maleic anhydride grafted PP decreased the creep ratio and increased the creep stiffness of the bituminous binder. Therefore, it is important to note that 5 wt.% WOPPr pyrolytic additive has a positive effect on both the low-temperature and high-temperature performance of pure bitumen.

### 3.12. Performance Grades (PGs)

The high-temperature PG of the binders can be determined according to the specifications by using the failure temperature at which the value of G*/sinδ is equalized to the 1.0 kPa [37,39,49]. Additionally, the low-temperature PG of the binders can be determined with the failure temperatures at which the m value is a minimum of 0.3 and the creep stiffness is a maximum of 300 MPa in accordance with the specifications [38,39,49]. The PG grades of bituminous binders have been determined as shown in Table 6.

According to the DSR [37] and BBR [38] test results, it was found that an increase in the high-temperature PG class of pure bitumen, from PG 64 to PG 76, was obtained with the addition of 5 wt.% WOPPr pyrolytic additive [Table 6]. Although this pyrolytic additive positively affected the low-temperature performance of neat bitumen [Figure 10], it was determined that it did not change the low-temperature PG class of pure bitumen [Table 6].

Comparing with the literature, one study [4] determined that 5% PP additive increased the high-temperature PG of pure bitumen from PG 64 to PG 70. Another study [8] reported that the PP additive also enhanced the PG of pure bitumen, and the other study [20] found that the 15% and 20% pyrolytic char powder of PP additive increased the PG of bitumen from PG 64 to PG 70.

## 4. Conclusions

In this paper, the effects of the WOPPr co-pyrolytic product on some rheological and physical characteristics of pure bitumen were investigated for the first time. The results are outlined below.

The WOPPr co-pyrolytic product was produced from the co-pyrolysis of WCO and PP at a certain ratio of 1:2 (WCO:PP) at 324 °C temperature. The WOPPr co-pyrolytic product removed from the reactor was found in a solid state at room temperature. This product was heated, and the liquid state of the WOPPr at 140 °C was used as an additive in PG 64-22 neat bitumen.

The PG 64-22 pure bitumen was modified with the WOPPr pyrolytic product at 160 °C temperature, 1200 rpm for 45 min. It can be said that the PG 64-22 pure bitumen can easily be modified with this pyrolytic product at the conditions described in this study.

It was found from the FTIR results of the modified binders that the intensity of the peak around 2357.69 cm^−1^ increased with the addition of this pyrolytic product.

The WOPPr co-pyrolytic additive hardened the pure bitumen consistency, decreased the penetration, and raised the softening point value. Additionally, this pyrolytic product increased the viscosity, enhanced the high-temperature performance and improved the rutting resistance of neat bitumen.

It was determined that the 3WOPPr and 5WOPPr-modified bituminous binders had suitable storage stability performance.

The increase in the high-temperature PG of pure bitumen, from PG 64 to PG 76, was obtained with the addition of the 5% WOPPr co-pyrolytic additive. This additive did not change the low-temperature PG of pure bitumen; however, it slightly enhanced and positively affected the low-temperature performance of neat bitumen.

Additionally, in this study, WCO was used during the production of the co-pyrolytic additive. Using waste materials, an easy modification of bitumen with novel additives and obtaining modified binders with better storage stabilities may have critical importance for enhancing the performance of bituminous binders and environmental and economic effects.

Therefore, for further studies, it may be suggested to investigate the effects and usability of different pyrolytic products from different materials by using the co-pyrolysis method for the modification of bituminous binders.

## Figures and Tables

**Table 1:** Some properties of 50/70 pure bitumen.

Properties	Results	Limits	Methods
Penetration (1/10 mm)	51.5	50–70	ASTM D5 [33]
Softening point (°C)	50	46–54	ASTM D36 [34]
Viscosity (cP) (135 °C)	467.5	max. 3000 cP	ASTM D4402 [35]
Mass change (%)	0.01	max. 1%	AASHTO T240 [36]
PG	64-22	G*/sinδ ≥ 1 kPa	AASHTO T315 [37]
		S ≤ 300 MPa	AASHTO T313 [38]
		m ≥ 0.300	AASHTO M320 [39]

**Table 2:** Some properties of the additive.

Additive *	Properties
Type of additive	Co-pyrolytic product
Co-pyrolysis conditions	324 °C, 110 min., WCO:PP (1:2)
Physical state	Solid (at room temperature)
Color	Dark brown
Melting peak temperature	140 °C

* The product produced in this study by using co-pyrolysis method as explained in detail in Section 3.1.

**Table 3:** Storage stability test results.

Additive	Softening Point °C		Softening Point	Penetration (1/10 mm)		Penetration
Content	Top Part	Bottom Part	Difference °C	Top Part	Bottom Part	Difference (1/10 mm)
Pure Bitumen	52.50	52.38	0.12	38.50	38.67	0.17
3WOPPr	56.00	55.50	0.50	32.33	32.57	0.24
5WOPPr	67.50	63.75	3.75	30.88	32.50	1.62
7WOPPr	88.50	72.00	16.50	29.83	31.17	1.34

**Table 4:** Viscosity values of bituminous binders.

Bituminous Binders	Pure Bitumen	5WOPPr
Viscosity (cP) (135 °C)	467.5	616.7

**Table 5:** Mass changes of binders.

Rolling Thin Film Oven Test		Pure Bitumen	5WOPPr
Mass Change (<1%)	%	0.01	0.22

**Table 6:** Performance grades (PGs) of bituminous binders.

Tests		Pure Bitumen	5WOPPr
DSR (G*/sinδ ≥ 1 kPa)	Failure Temperature (°C)	69.1	81.9
BBR (S ≤ 300 MPa, m ≥ 0.300)	Failure Temperature (°C)	−12	−12
PG Grades		64-22	76-22

## Data Availability

Dataset available on request from the author.
